# *Mycoplasma genitalium* and Other Reproductive Tract Infections in Pregnant Women, Papua New Guinea, 2015–2017

**DOI:** 10.3201/eid2703.201783

**Published:** 2021-03

**Authors:** Michelle J.L. Scoullar, Philippe Boeuf, Elizabeth Peach, Ruth Fidelis, Kerryanne Tokmun, Pele Melepia, Arthur Elijah, Catriona S. Bradshaw, Glenda Fehler, Peter M. Siba, Simon Erskine, Elisa Mokany, Elissa Kennedy, Alexandra J. Umbers, Stanley Luchters, Leanne J. Robinson, Nicholas C. Wong, Andrew J. Vallely, Steven G. Badman, Lisa M. Vallely, Freya J.I. Fowkes, Christopher Morgan, William Pomat, Brendan S. Crabb, James G. Beeson, Healthy Mothers Healthy Babies Study Team

**Affiliations:** Burnet Institute, Melbourne, Victoria, Australia (M.J.L. Scoullar, P. Boeuf, E. Peach, R. Fidelis, K. Tokmun, P. Melepia, E. Kennedy, A.J. Umbers, S. Luchters, L.J. Robinson, F.J.I. Fowkes, C. Morgan, B.S. Crabb, J.G. Beeson);; Burnet Institute, Kokopo, Papua New Guinea (M.J.L. Scoullar, P. Boeuf, E. Peach, R. Fidelis, K. Tokmun, P. Melepia, E. Kennedy, A.J. Umbers, S. Luchters, L.J. Robinson, F.J.I. Fowkes, C. Morgan, B.S. Crabb, J.G. Beeson);; University of Melbourne, Melbourne (M.J.L Scoullar, P. Boeuf, C.S. Bradshaw, L.J. Robinson, F.J.I. Fowkes, C. Morgan, B.S. Crabb, J.G. Beeson);; University of Papua New Guinea, Port Moresby, Papua New Guinea (A. Elijah);; Melbourne Sexual Health Centre, Melbourne (C.S. Bradshaw, G. Fehler);; Monash University, Melbourne (C.S. Bradshaw, S. Luchters, L.J. Robinson, N.C. Wong, F.J.I. Fowkes, C. Morgan, B.S. Crabb, J.G. Beeson);; Papua New Guinea Institute of Medical Research, Goroka, Papua New Guinea (P.M. Siba, L.J. Robinson, A. Vallely, L.M. Vallely, W. Pomat);; SpeeDx Pty Ltd, Sydney, New South Wales, Australia (S. Erskine, E. Mokany);; Aga Khan University, Nairobi, Kenya (S. Luchters);; Ghent University, Ghent, Belgium (S. Luchters);; University of New South Wales, Sydney (A. Vallely, S.G. Badman, L.M. Vallely);; James Cook University, Townsville, Queensland, Australia (L.M. Vallely)

**Keywords:** sexually transmitted infections, *Mycoplasma genitalium*, *Chlamydia trachomatis*, *Neisseria gonorrhoeae*, *Trichomonas vaginalis*, *Treponema pallidum*, pregnant women, Papua New Guinea, pregnancy, bacterial vaginosis, vulvovaginal candidiasis, bacteria, fungi, reproductive tract infections

## Abstract

Much about the range of pathogens, frequency of coinfection, and clinical effects of reproductive tract infections (RTIs) among pregnant women remains unknown. We report on RTIs (*Mycoplasma genitalium*, *Chlamydia trachomatis*, *Neisseria gonorrhoeae*, *Trichomonas vaginalis*, *Treponema pallidum* subspecies *pallidum*, bacterial vaginosis, and vulvovaginal candidiasis) and other reproductive health indicators in 699 pregnant women in Papua New Guinea during 2015–2017. We found *M. genitalium*, an emerging pathogen in Papua New Guinea, in 12.5% of participants. These infections showed no evidence of macrolide resistance. In total, 74.1% of pregnant women had >1 RTI; most of these infections were treatable. We detected sexually transmitted infections (excluding syphilis) in 37.7% of women. Our findings showed that syndromic management of infections is greatly inadequate. In total, 98.4% of women had never used barrier contraception. These findings will inform efforts to improve reproductive healthcare in Papua New Guinea.

Reproductive tract infections (RTIs), including sexually transmitted infections (STIs), are preventable and often curable health conditions. Public health officials consider *Chlamydia trachomatis*, *Neisseria gonorrhoeae*, *Trichomonas vaginalis*, and *Treponema pallidum* subspecies *pallidum* infections to be curable diseases. An estimated 376.4 million new cases of these 4 infections occur globally in adults each year; the World Health Organization Western Pacific Region has the highest number of annual new cases, estimated at 142 million (*1*–*3*). Other RTIs, such as bacterial vaginosis (BV) and vulvovaginal candidiasis (VVC) caused by *Candida* *albicans*, are also common. However, global estimates for these diseases are less certain because of differing diagnostic methodologies for BV (*4*) and prevalence of commensal *C. albicans*. Current estimates suggest that 8%–51% of pregnant women have BV (*5*); 20%–30% of asymptomatic and 40% of symptomatic women have vaginal *C. albicans* infections (*6*). RTIs can cause substantial pain and discomfort and some patients might experience debilitating stigma from their families and communities (*7*). Possible complications include pelvic inflammatory disease, infertility, and increased risk for other STIs. In pregnant women, RTIs can cause miscarriage, stillbirth, preterm birth, or neonatal death, as well as serious neonatal conditions such as blindness, congenital malformations, and lifelong disability (*1*,*8*,*9*).

*Mycoplasma genitalium* is increasingly understood to be a major cause of poor sexual health and is associated with pelvic inflammatory disease, cervicitis, miscarriage, and preterm birth (*10*,*11*). Limited data exists on *M. genitalium* prevalence, although estimates range from <1.0% in the general adult population to 15.9% in groups at high risk, such as female commercial sex workers (*12*,*13*). In pregnant women, estimates range from 0.7% in the United Kingdom (*14*) to 11.9% in the Solomon Islands (*15*). During 2010–2019, global macrolide resistance to *M. genitalium* increased from 10% to >50% (*16*). In many regions, the prevalence of *M. genitalium* and its susceptibility to antimicrobial drugs is unknown.

Papua New Guinea is a country in the southwestern Pacific Ocean with >8.5 million persons (*17*). Poor pregnancy outcomes are common in this country. Estimates are imprecise because of weaknesses in vital registry systems, but <50% of women give birth with a skilled birth attendant (*18*). Ultrasound machines for gestational age assessment are largely inaccessible because of scarcity, cost, and location. The estimated prevalence of low birthweight (weight <2.5 kg) ranges from 10%–24% and preterm birth from 7%–18% (*19*). Papua New Guinea has a high perinatal death rate of 17 deaths/1,000 pregnancies (*19*). Curable STIs are common; rates of *C. trachomatis*, *N. gonorrhoeae*, *T. vaginalis*, and *T. pallidum* infections exceed those of other high-prevalence regions such as sub-Saharan Africa (*1*,*20*). However, little to no data exists on the prevalence of *M. genitalium* in Papua New Guinea. We evaluated the prevalence of *M. genitalium* and other RTIs among pregnant women attending antenatal clinics in the East New Britain (ENB) province of Papua New Guinea. We also investigated molecular markers of resistance in clinical samples from these patients. We investigated the relationships between different RTIs, factors associated with infection, and analyzed the diagnostic accuracy of syndromic management guidelines.

## Materials and Methods

### Study Site and Population

We studied cross-sectional baseline data from 699 pregnant women attending their first antenatal clinic. Study participants were enrolled in Healthy Mothers Healthy Babies, a prospective cohort study undertaken at 5 health facilities in 3 of the 4 districts of ENB. The study sites included the hospitals in the 2 major urban areas and the 3 largest rural health centers of ENB. Members of the largest ethnic group, the Tolai, access all facilities; members of the second largest ethnic group, the Baining, predominantly access Kerevat Rural Hospital, the government-operated rural facility. Enrollment in the Healthy Mothers Healthy Babies cohort, and thus this study, occurred during March 2015–June 2017. Women >16 years of age who were living in the facilities’ catchment area and attending clinic for the first time in the current pregnancy, regardless of gestational age, were eligible to participate. At each site, women were randomly selected through a dice roll. After the women underwent eligibility screening and provided informed consent, they completed a questionnaire administered by a trained research officer. We collected sociodemographic and clinical information through the questionnaire and patient-held medical records. We obtained urine, capillary finger prick blood, self-collected vaginal swab, and venous blood samples. We communicated all abnormal results available at the point of care, such as results of the urine dipstick and syphilis, malaria, and hemoglobin assays, to the participant and the healthcare provider.

### Study Procedures

Health facility staff provided routine antenatal care, including intermittent preventive treatment in pregnancy for malaria, syndromic management for vaginal discharge (Appendix), iron and folate supplementation, voluntary counselling and testing for HIV using Alere Determine HIV-1/2 (Abbott, https://www.abbott.com), and point-of-care syphilis testing using Alere Determine Syphilis TP (Abbott), in accordance with national guidelines (*21*,*22*). At the beginning of the study period, the participating healthcare facilities conducted syphilis testing. However, interruptions in stock supply nationally led to fewer women being tested for syphilis. The research team subsequently supplied and conducted testing for study participants. Stock interruptions of HIV testing materials also occurred; however, our research team was not qualified for voluntary counselling and testing and did not have ethics approval to conduct HIV testing.

Each participant provided 2 self-collected vaginal swab samples: 1 GeneXpert vaginal/endocervical swab (Cepheid, https://www.cepheid.com), which was placed directly into its transport medium, and 1 Copan flocked swab (Copan Diagnostics, Inc., https://www.copanusa.com), which was first used to prepare a vaginal smear on a slide for microscopy, and then placed in 1.0 mL Copan Universal Transport Medium (Copan Diagnostics, Inc.) specific for bacterial STIs. The number of vaginal swabs and smears available for diagnosis varied because of occasional reluctance to provide a swab, quality of vaginal smear, and availability of GeneXpert testing cartridges. Each woman self-collected a urine sample in a sterile container. All specimens were stored in a chilled box at 2°C–7°C for the remainder of clinic day, then stored at 2°C–7°C or −20°C until tested.

### Laboratory Methods

We used the GeneXpert molecular platform (Cepheid) to test vaginal and urine specimens for *C. trachomatis*, *N. gonorrhoeae*, and *T. vaginalis* at the Burnet Institute/Papua New Guinea Institute of Medical Research laboratory at St. Mary’s Hospital Vunapope (Kokopo, Papua New Guinea). *M. genitalilum* and resistance mutations were detected by quantitative PCR (ResistancePlus MG kit, SpeeDx Pty Ltd, https://plexpcr.com). Gram-stained vaginal smears were read by an experienced microscopist at the Melbourne Sexual Health Centre (Melbourne, Victoria, Australia) (Appendix). 

### Data Management and Statistical Analysis

Researchers interviewed participants and documented their responses using electronic tablets. We employed stringent data management protocols (Appendix).

The questionnaire included details about the enrollment clinic, participant characteristics at enrollment, and relevant obstetric history (Appendix). This study produced prevalence estimates of *M. genitalium*, *C. trachomatis*, *N. gonorrhoeae*, *T. vaginalis*, *T. pallidum*, BV, and VVC among pregnant women in Papua New Guinea. We used logistic regression to assess the association between patient characteristics and STIs, including *C. trachomatis*, *N. gonorrhoeae*, *T. vaginalis*, and *M. genitalium*. We included all variables of interest in the univariable analysis. The multivariable model retained variables associated with the outcome at p<0.10 in the univariable analysis. We also analyzed the effectiveness of syndromic management guidelines using the standard question about current symptoms compared with an alternative question about symptoms experienced during the current pregnancy.

### Ethics Considerations

All participants provided individual written, informed consent. Ethics approval was provided from the Medical Research Advisory Committee of the Papua New Guinea National Department of Health (approval no. 14.27), the Papua New Guinea Institute of Medical Research Institutional Review Board (approval no. 1114), and the Human Research Ethics Committee of the Alfred Hospital in Australia (approval no. 348/14). Provincial approval was obtained from the East New Britain Provincial Executive Committee and participating facilities. A series of community engagement meetings provided broader community support and assent for the study.

## Results

We enrolled 699 pregnant women at 5 antenatal clinics in ENB. The median maternal age was 26 years (interquartile range [IQR] 22–30 years), 25.3% (177/699) of women were primigravida, 95.1% (663/697) were married or lived with a partner, and 46.5% (325/698) had only completed primary school (Table 1). In total, 82.5% (569/690) of women had never used a modern method of contraception; only 11 (1.6%) women had ever used a condom for men or women.

**Table 1:** Sociodemographic characteristics and obstetric history of women at first antenatal clinic visit in East New Britain, Papua New Guinea, 2015–2017*

Characteristic	Value
Total	699 (100.0)
Sociodemographic	
Enrollment clinic	
St. Mary’s Hospital Vunapope	184 (26.3)
Nonga General Hospital	83 (11.9)
Kerevat Rural Hospital	125 (17.9)
Napapar Health Centre	158 (22.6)
Paparatava Health Centre	149 (21.3)
Clinic administration	
Government	208 (29.8)
Catholic Health Services	491 (70.2)
Clinic setting	
Urban	342 (48.9)
Rural	357 (51.1)
Age, y†	
Median age (IQR), range	26 (22–30), 16–49
16–24	275 (39.7)
25–34	334 (48.3)
>35	83 (12.0)
Highest level of education completed‡	
Primary: grade 8 or less	325 (46.6)
High school: grades 9, 10	177 (25.4)
Secondary, vocational, or tertiary	196 (28.1)
Employment status	
Not employed	531 (76.0)
Employed (paid work) or student	168 (24.0)
Province of birth	
East New Britain	578 (82.7)
Other	121 (17.3)
Religion‡	
Catholic	345 (49.4)
Other	353 (50.6)
Marital status§	
Married or cohabiting	663 (95.1)
Single, separated, or widowed	34 (4.9)
Polygamy¶	
1 wife	583 (88.1)
>1 wife	79 (11.9)
Median household monthly expenditure, kina (SD)#	150 (50–300)
Median cost of antenatal care visit, kina (SD)**	4 (2–20)
Family planning‡‡	
Never used modern method	569 (82.5)
Has used modern method	121 (17.5)
Maternal health parameters at first antenatal clinic	
Gravidity	
Primigravidae	177 (25.3)
Multigravidae: 2nd–4th pregnancy	384 (54.9)
Grandmulti: ≥5th pregnancy	138 (19.7)
Abnormal vaginal discharge	
At any time during pregnancy‡	135 (19.3)
Currently§	98 (14.0)
Smoking§	
Never smoked	427 (61.3)
Stopped when pregnant	241 (34.6)
Current smoker	29 (4.2)
Previous pregnancy outcomes	
Median age at first pregnancy, y (IQR)††	21 (19–24)
History pregnancy loss‡‡	
Miscarriage	46 (8.8)
Abortion	1 (0.2)
Stillbirth	16 (3.1)
Partner information	
Partner's employment status§§	
Unemployed	269 (39.6)
Employed in paid work	411 (60.4)
Partner attending first antenatal clinic¶¶	
No	571 (82.3)
Yes	123 (17.7)

*Values are no. (%) except as indicated. N = 699, except as indicated. †Missing age data for 9 women, missing age group for 7 women. ‡Missing data for 1 woman. §Missing data for 2 women. ¶Missing data for 37 women. #Missing data for 17 women. 1.00 Papua New Guinean kina = 0.28 US dollar. **Missing data for 9 women. 1.00 Papua New Guinean kina = 0.28 US dollar. ††Missing data for 7 women. ‡‡Out of 520 women who had previous pregnancies. §§Missing data for 19 women. ¶¶Missing data for 5 women.

### High Burden of RTIs During Pregnancy

The total number of women tested for each pathogen varied as detailed in Methods. Of the 699 women enrolled, 12.5% (78/625; 95% CI 10.0%–15.3%) had *M. genitalium* infections. We found no evidence of macrolide-resistant mutations (Table 2). Among the samples tested, 19.1% (122/640; 95% CI 16.1%–22.3%) of women had *C. trachomatis* infections, 5.5% (35/640; 95% CI 3.8%–7.5%) had *N. gonorrhoeae* infections; and 20.1% (117/581; 95% CI 17.0%–23.7%) of tested samples were positive for *T. vaginalis*. Lifetime exposure to syphilis was extremely high: 18.1% (79/437; 95% CI 14.6%–22.0%) of samples were positive by *T. pallidum *serologic testing. Among the 653 vaginal smears available for microscopy, BV prevalence was 26.0% (170/653; 95% CI 22.7%–29.6%) and VVC prevalence was 37.5% (245/653; 95% CI 33.8%–41.4%). Facility-based HIV rapid test results were available for 205 women, of whom 2 (0.98%) were HIV-positive.

**Table 2:** Prevalence of reproductive tract infections among pregnant women in East New Britain, Papua New Guinea, 2015–2017*

Reproductive tract infection	Tested	Frequency	Prevalence, % (95% CI)
No current RTI†	467	121	25.9 (22–30.1)
No current STI‡	485	302	62.3 (57.8–66.6)
*Mycoplasma genitalium*	625	78	12.5 (10–15.3)
*Chlamydia trachomatis*	640	122	19.1 (16.1–22.3)
*Neisseria gonorrhoeae*	640	35	5.5 (3.8–7.5)
*Trichomonas vaginalis*	581	117	20.1 (16.9–23.6)
Syphilis§	437	79	18.1 (14.6–22)
Bacterial vaginosis	653	170	26 (22.7–29.6)
Vulvovaginal candidiasis	653	245	37.5 (33.8–41.4)
Co-infections			
>1 Current RTI	467	346	74.1 (69.9–78)
>1 Current STI	485	183	37.7 (33.4–42.2)
			
>1 MG, CT, NG, TV, or syphilis infection	302	144	47.7 (41.9–53.5)
>1 MG, CT, NG, TV, or BV infection	467	250	53.5 (48.9–58.1)
>1 Infection diagnosed by GeneXpert¶	546	175	32.1 (28.2–36.1)
>1 Vaginal infection#	542	362	66.8 (62.6–70.7)
>1 BV or VVC infection	653	376	57.6 (53.7–61.4)
			
Multiple current STIs			
2	661	75	11.3 (9–14)
3	536	15	2.8 (1.6–4.6)

*Participants result included only if they had all tests done for each of the infections within group of RTIs. BV, bacterial vaginosis; CT, *Chlamydia trachomatis*; MG, *Mycoplasma genitalium*; NG, *Neisseria gonorrhoeae*; RTI, reproductive tract infection; STI, sexually transmitted infection; TV, *Trichomonas vaginalis*; VVC, vulvovaginal candidiasis. RTIs include MG, CT, NG, TV, BC, and VVC (syphilis not included). STIs include MG, CT, NG, TV (syphilis not included). §Diagnosed with Alere Determine Syphilis TP (Abbott, https://www.abbott.com). ¶CT, NG, and TV infections diagnosed with GeneXpert (Cepheid, https://www.cepheid.com). #Vaginal infections include BV, TV, and VVC.

Among women for whom all results were available, most (74.1%; 346/467) had >1 RTI (i.e., BV, VVC, *M. genitalium*, *C. trachomatis*, *N. gonorrhoeae*, or *T. vaginalis*) at the time of screening; 37.7% (183/485) had >1 curable STI (i.e., *M. genitalium*, *C. trachomatis*, *N. gonorrhoeae*, or *T. vaginalis*) at the time of screening. Among the women who were tested, 32.1% (175/546) had an STI diagnosed using GeneXpert (*C. trachomatis*, *N. gonorrhoeae*, or *T. vaginalis*), 11.3% (75/661) had >2 concurrent STIs, 2.8% (15/536) of women had >3 coinfections, and 1 woman had 4 STIs.

### Associations between Infections

**Figure 1:**
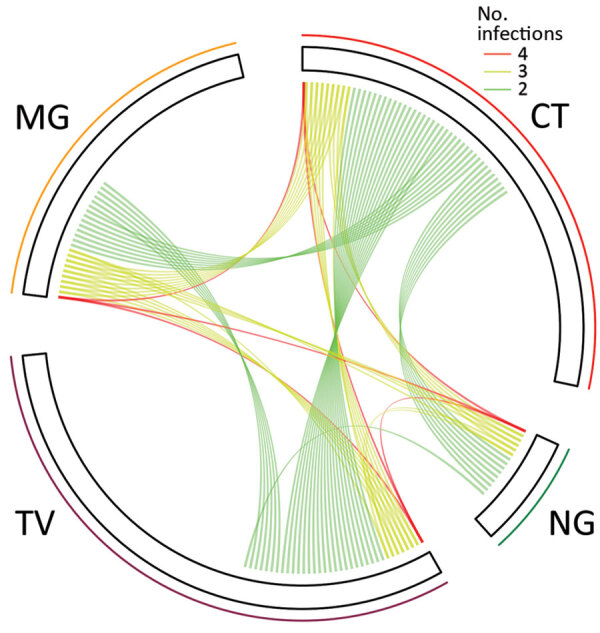
Relationships among sexually transmitted infections in pregnant women, East New Britain, Papua New Guinea, 2015–2017. Each line indicates >2 concurrent infections in 1 participant. The length of each sector corresponds to the number of monoinfections. MG, *Mycoplasma genitalium*; CT, *Chlamydia trachomatis*; NG, *Neisseria gonorrhoeae*; TV, *Trichomonas vaginalis*.

Of the 78 women with *M. genitalium* infections, 28 (35.9%) had >1 concurrent STI detected: 20 (25.6%) had *C. trachomatis* infections, 13 (16.7%) had *T. vaginalis* infections, and 6 (7.7%) had *N. gonorrhoeae* infections (Figure 1; Appendix Table 2). Co-infections were most frequent among women with *N. gonorrhoeae* infections (80%; 28/35); most women with *N. gonorrhoeae* infections also had *C. trachomatis* infections (71.4%; 25/35), *T. vaginalis* infections (22.8%; 8/35), or *M. genitalium* infections (17.1%; 6/35). We did not consider syphilis in estimates of coinfections because the syphilis test did not distinguish between current or previous infection. Of 170 women with BV, 40.6% (69/170) had a co-infection; the most common were *C. trachomatis* (24.1%; 41/170), *T. vaginalis* (12.3%; 21/170), *M. genitalium* (12.3%; 21/170), and *N. gonorrhoeae* (7.6%; 13/170) (Appendix Table 3).

### Relationship between Abnormal Vaginal Discharge and Infection

We compared the infections of women with current abnormal vaginal discharge (as defined by national treatment guidelines) with those who had abnormal vaginal discharge currently or at any time in pregnancy before their first antenatal clinic visit (Table 3). A total of 98 women (14.1%; 98/697) had current symptoms (i.e., abnormal vaginal discharge) that would have prompted treatment according to syndromic management guidelines (2 women did not answer this question). An additional 37 women did not have abnormal vaginal discharge at the time of the screening but had experienced it earlier in the pregnancy. According to the national treatment guidelines, these women would not normally receive treatment.

**Table 3:** Screening question for RTIs in pregnant women, East New Britain, Papua New Guinea, 2015–2017*

Category (*22*)	Screening question as per syndromic management guidelines: Do you currently have any abnormal vaginal discharge?				Alternative question: Have you experienced any abnormal vaginal discharge earlier in the pregnancy or now?		
	No	Yes	Total		No	Yes	Total
Total	599 (85.9)	98 (14.1)	697		563 (80.7)	135 (19.3)	698
Reproductive tract infection							
No current RTI†	112 (93.3)	8 (6.7)	120		108 (89.3)	13 (10.7)	121
No current STI‡	265 (88.0)	36 (12.0)	301		249 (82.5)	53 (17.5)	302
*Mycoplasma genitalium*	66 (84.6)	12 (15.4)	78		61 (78.2)	17 (21.8)	78
*Chlamydia trachomatis*	98 (80.3)	24 (19.7)	122		90 (73.8)	32 (26.2)	122
*Neisseria gonorrhoeae*	28 (80.0)	7 (20.0)	35		26 (74.3)	9 (25.7)	35
*Trichomonas vaginalis*	94 (80.3)	23 (19.7)	117		83 (70.9)	34 (29.1)	117
Syphilis§	68 (86.1)	11 (13.9)	79		65 (82.3)	14 (17.7)	79
Bacterial vaginosis	146 (85.9)	24 (14.1)	170		136 (80.0)	34 (20.0)	170
Vulvovaginal candidiasis	199 (81.2)	46 (18.8)	245		182 (74.3)	63 (25.7)	245
Co-infections							
>1 Current RTI	292 (84.4)	54 (15.6)	346		268 (77.5)	78 (22.5)	346
>1 Current STI	154 (84.2)	29 (15.8)	183		141 (77.0)	42 (23.0)	183
>1 Infection diagnosed by GeneXpert¶	141 (80.6)	34 (19.4)	175		127 (72.6)	48 (27.4)	175
>1 Vaginal infection#	298 (82.3)	64 (17.7)	362		271 (74.9)	91 (25.1)	362
>1 BV or VVC infection	314 (83.5)	62 (16.5)	376		290 (77.1)	86 (22.9)	376
Any 2 current STIs	58 (77.3)	17 (22.7)	75		53 (70.7)	22 (29.3)	75

*Values are frequency, no. (%). Missing data for 1 woman who responded yes to the alternative question had a missing response to the standard question. BV, bacterial vaginosis; CT, *Chlamydia trachomatis*; MG, *Mycoplasma genitalium*; NG, *Neisseria gonorrhoeae*; RTI, reproductive tract infection; STI, sexually transmitted infection; TV, *Trichomonas vaginalis*; VVC, vulvovaginal candidiasis. RTIs include MG, CT, NG, TV, BC, and VVC (syphilis not included). STIs include MG, CT, NG, TV (syphilis not included). §Diagnosed with Alere Determine Syphilis TP (Abbott, https://www.abbott.com). ¶CT, NG, and TV infections diagnosed with GeneXpert (Cepheid, https://www.cepheid.com). #Vaginal infections include BV, TV, and VVC.

Most STIs were asymptomatic and neither criteria (current abnormal vaginal discharge vs. current or previous abnormal vaginal discharge during this pregnancy) performed well as a marker of infection. Of those women with a detected STI, 84.1% (154/183) had no current symptoms and 77.0% (141/183) had not experienced symptoms during their current pregnancy. Conversely, 12.0% (36/301) of uninfected women had current symptoms and 17.6% (53/302) had experienced symptoms during their current pregnancy. Of those with *M. genitalium* infection, only 12 women (15.4%;12/78) would have been treated according to syndromic management guidelines used by Papua New Guinea.

**Figure 2:**
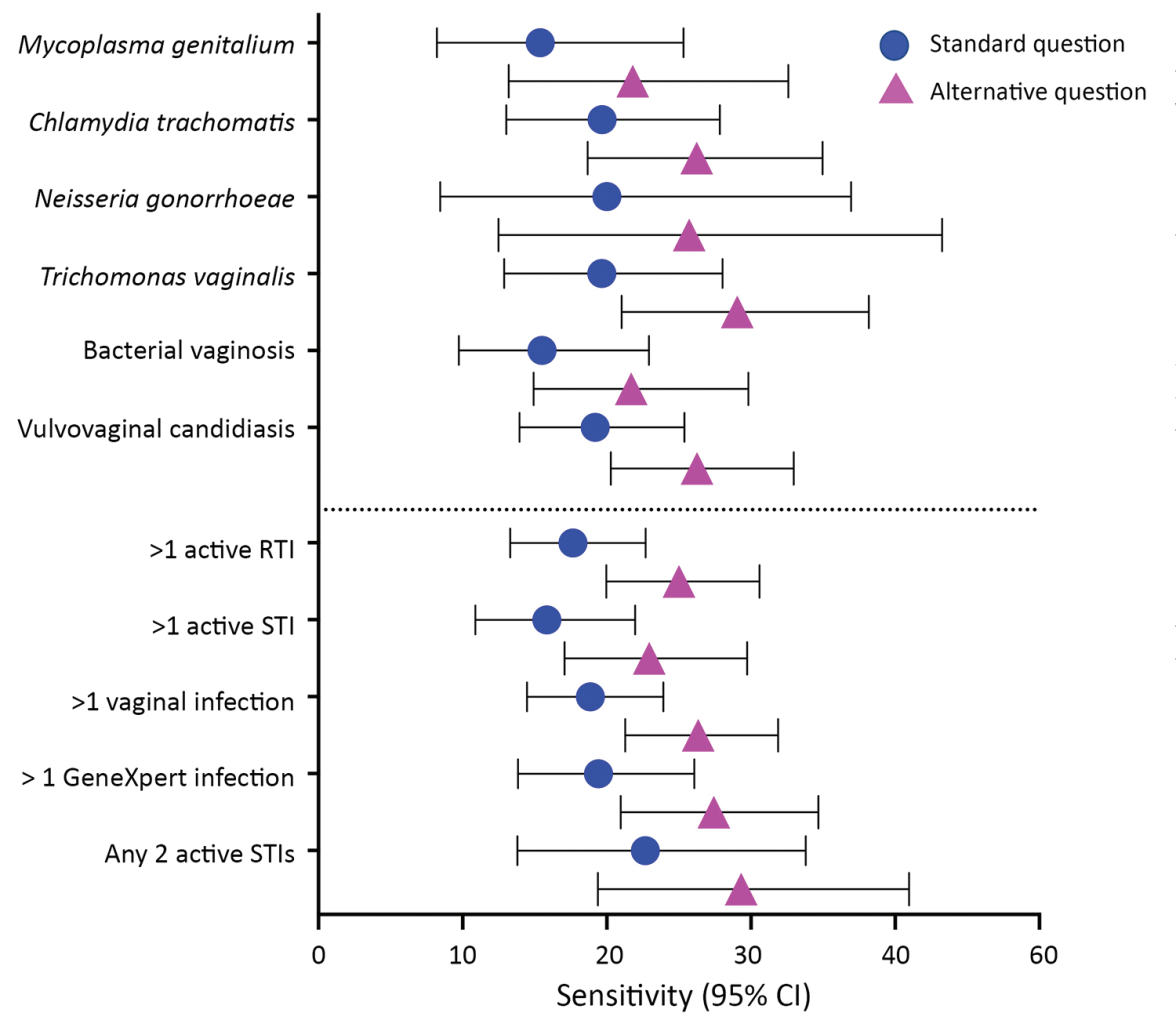
Sensitivity of syndromic management for sexually transmitted infections in pregnant women, East New Britain, Papua New Guinea, 2015–2017. Participants answered the standard question according to Papua New Guinea national guidelines “Do you currently have any abnormal vaginal discharge?” or the alternative question “Have you experienced any abnormal vaginal discharge earlier in the pregnancy or now?” (*22*). *Chlamydia trachomatis*, *Neisseria gonorrhoeae*, and *Trichomonas vaginalis* infections diagnosed with GeneXpert (Cepheid, https://www.cepheid.com). RTI, reproductive tract infection; STI, sexually transmitted infection.

Asking whether women had any symptoms during their current pregnancy was consistently more sensitive than asking about current symptoms as per the standard diagnostic question (Figure 2); however, the sensitivity of both questions was <30% for all individual or collective pathogens. The alternative question was less specific for >1 current STI (82.5% [p = 0.15] vs. 88% [p = 0.22]; Appendix Table 4). The alternative question was best able to identify women with *T. vaginalis* infection (p<0.01) and VVC (p<0.01) (Appendix Table 4); however, this question still missed most infections.

### Factors Associated with Curable STIs

We did not identify any factors in the univariable (Appendix Table 5) or multivariable (Table 4) analysis that were associated with an increased odds of *M. genitalium* infection. The univariable analysis showed that women who were younger, in their first pregnancy, employed, single or separated, had never used a modern method of contraception, or had abnormal vaginal discharge at any time in their current pregnancy were at increased risk for certain STIs, to varying degrees of statistical significance. In the multivariable analysis, primigravida women and those 16–24 years of age had higher odds for *C. trachomatis* infection (adjusted odds ratio [aOR] 2.17, 95% CI 1.29–3.64 [p<0.01], and aOR 3.39, 95% CI 1.24–9.28 [p = 0.02], respectively). Primigravida women also had higher odds for *N. gonorrhoeae* infection (aOR 4.33, 95% CI 1.74–10.75; p<0.01). Women 16–24 years of age had increased odds for testing positive for >1 STI compared with women in other age groups (aOR 2.45, 95% CI 1.17–5.16; p = 0.02).

**Table 4:** Multivariable analysis of factors associated with current sexually transmitted infections in pregnant women, East New Britain, Papua New Guinea, 2015–2017*

Characteristic	Sexually transmitted infection, aOR (95% CI); p value				
	*Mycoplasma genitalium*	*Chlamydia trachomatis*	*Neisseria gonorrhoeae*	*Trichomonas vaginalis*	>1 infection
Clinic					
Vunapope	Referent	Referent	Referent	Referent	Referent
Nonga	0.68 (0.28–1.62); 0.38	0.88 (0.43–1.78); 0.72	2.35 (0.76–7.32); 0.14	0.91 (0.42–1.97); 0.82	0.84 (0.43–1.63); 0.61
Kerevat	0.9 (0.43–1.88); 0.77	0.55 (0.28–1.09); 0.09	1.03 (0.3–3.5); 0.97	0.84 (0.41–1.72); 0.62	0.58 (0.31–1.11); 0.10
Napapar	0.76 (0.37–1.57); 0.46	0.9 (0.5–1.62); 0.73	0.99 (0.31–3.15); 0.98	1.09 (0.6–1.99); 0.77	0.64 (0.36–1.13); 0.12
Paparatava	0.86 (0.43–1.73); 0.68	0.86 (0.47–1.57); 0.62	2.04 (0.7–5.95); 0.19	1.08 (0.59–1.99); 0.79	1.01 (0.58–1.75); 0.97
Age, y					
>35	Referent	Referent	Referent	Referent	Referent
25–34	0.76 (0.34–1.69); 0.50	2.47 (0.94–6.52); 0.07	1.01 (0.2–4.98); 0.99	1.85 (0.78–4.37); 0.16	1.7 (0.85–3.38); 0.13
16–24	1.21 (0.52–2.82); 0.66	3.39 (1.24–9.28); 0.02	1.86 (0.36–9.63); 0.46	2.31 (0.93–5.7); 0.07	2.45 (1.17–5.16); 0.02
Gravidity					
Multigravida	Referent	Referent	Referent	Referent	Referent
Primigravida	0.87 (0.46–1.65); 0.67	2.17 (1.29–3.64); <0.01	4.33 (1.74–10.75); <0.01	1.09 (0.62–1.92); 0.75	1.45 (0.87–2.42); 0.15
Marital status					
Married/cohabiting	Referent	Referent	Referent	Referent	Referent
Single/separated	1.06 (0.34–3.3); 0.92	1.31 (0.54–3.13); 0.55	0.44 (0.09–2.23); 0.32	4.48 (1.9–10.55); <0.01	1.6 (0.61–4.21); 0.34
Vaginal discharge					
No symptoms	Referent	Referent	Referent	Referent	Referent
Abnormal discharge (current or before first antenatal clinic)	1.17 (0.63–2.15); 0.62	1.29 (0.78–2.14); 0.33	1.45 (0.6–3.53); 0.41	1.56 (0.94–2.59); 0.09	1.29 (0.79–2.11); 0.31
Has used modern contraception					
Yes	Referent	Referent	Referent	Referent	Referent
No	1.82 (0.82–4.08); 0.14	1.04 (0.56–1.95); 0.90	0.77 (0.23–2.59); 0.67	1.27 (0.66–2.43); 0.47	1.17 (0.67–2.05); 0.57
Employment status					
Unemployed	Referent	Referent	Referent	Referent	Referent
Employed	0.89 (0.49–1.62); 0.71	1.27 (0.79–2.05); 0.32	2.66 (1.24–5.71); 0.01	0.96 (0.57–1.62); 0.89	1.29 (0.81–2.06); 0.28
Urine nitrite					
Trace	0.49 (0.11–2.12); 0.34	0.78 (0.25–2.43); 0.667	0.68 (0.08–5.55); 0.72	0.34 (0.07–1.61); 0.17	0.35 (0.11–1.11); 0.08
Positive	1.32 (0.58–3); 0.50	1.88 (0.94–3.74); 0.07	1.6 (0.5–5.09); 0.43	1.29 (0.6–2.76); 0.51	1.26 (0.62–2.58); 0.53
Fever during pregnancy					
No	Referent	Referent	Referent	Referent	Referent
Yes (before first antenatal clinic)	1.15 (0.66–1.99); 0.63	0.75 (0.46–1.24); 0.26	0.69 (0.29–1.65); 0.41	1.58 (0.99–2.53); 0.05	1 (0.63–1.57); 0.99

*aOR, adjusted odds ratio.

## Discussion

We confirmed that *M. genitalium* is widespread among pregnant women in Papua New Guinea, which has one of the highest prevalence rates of this infection globally. We did not find evidence of macrolide resistance. The high prevalence of *M. genitalium* (12.5%) among pregnant women suggests an estimated 13,000 (95% CI 10,342–15,823) current cases among women of reproductive age in the province (Appendix). In addition, we provide contemporary data on RTIs in pregnant women from the New Guinea Islands region of Papua New Guinea; the most recent report on the subject is >20 years old (*23*). Our study indicates that >1 in 2 (53.5%) pregnant women in ENB have a treatable RTI (including BV, STI, or both) known to cause harmful sexual and reproductive health outcomes. These RTIs are not usually detectable by the syndromic management practices described in the national health guidelines of PNG. This high prevalence of poor sexual and reproductive health has major national and regional public health significance.

No global surveillance system for *M. genitalium* currently exists (*24*). Different detection methods have varying levels of sensitivity, limiting scientific understanding of its epidemiology. High-income countries report rates of *M. genitalium* infection ranging from 0.3%–3.3% (*11*,*13*,*25*) in the general population, with higher estimates in certain populations (*26*,*27*). Fewer data are available from low- and middle-income countries (LMICs) but prevalence appears to be higher, ranging from 3% in the general population in Tanzania (*13*) to 8%–9% in Honduras and South Africa (*13*,*28*). The highest prevalence has been reported among sex workers: 16% in Kenya (*29*) and 26% in Uganda (*30*). Data on *M. genitalium* infection among pregnant women remains limited despite the disease’s association with adverse pregnancy outcomes (*26*); available estimates range from 0.7%–0.9% in the United Kingdom and France (*14*,*31*) to 6.2% in Guinea-Bissau (*32*) and 11.9% in the Solomon Islands (*15*). More data on the prevalence and consequences of *M. genitalium* infection among pregnant women are needed.

Regional data on *M. genitalium* in LMICs are limited. One study from the Solomon Islands examined the effects of mass drug administration (MDA) using 1 g of oral azithromycin for eliminating ocular *C. trachomatis* on *M. genitalium* infection rates (*15*). Before MDA, the study found an 11.9% (95% CI 8.3%–16.6%; n = 236) prevalence of *M. genitalium* among pregnant women. After MDA, the prevalence remained high at 10.9% with no evidence of macrolide resistance. However, only 5 of the 28 *M.* *genitalium*–positive women in the post-MDA group had received azithromycin (*15*). 

In this study, the lack of macrolide resistance among *M. genitalium* infections in pregnant women warrants further exploration. Macrolide susceptibility might reflect a population’s lack of exposure to this class of antimicrobial drugs. However, macrolides are used widely in Papua New Guinea (*22*,*33*) and are available without prescription (although over-the-counter macrolides are more expensive than their prescribed counterparts).

We observed a prevalence of curable STIs substantially greater than in most settings included in the 2016 global estimates of curable STIs (*3*). The 32.1% observed prevalence of >1 current STI diagnosable by GeneXpert is less than the 42.7% reported in a study of antenatal clinics from 3 mainland provinces of Papua New Guinea in 2014 (*20*), but similar to the 33.7% prevalence among pregnant women in Madang Province in 2012 (*34*). We found a 19.1% prevalence of *C. trachomatis* infection among pregnant women, consistent with reports from other provinces (22.9% in the Eastern Highlands, Hela, and Central provinces [*20*] and 20.0% in the Milne Bay province [*35*]) and the neighboring Solomon Islands (20.3%) (*36*). Similarly, Papua New Guinea and Solomon Islands have the highest reported rates of *N. gonorrhoeae* among pregnant women (5.1%–14.2%) (*34*,*36*–*38*) in the world. In addition, 2 studies from South Africa also report very high rates of *N. gonorrhoeae*: 10.1% among patients in a primary care setting (*39*) and 6.4% among pregnant women (*40*).

Risk factors for different STIs identified in this study (e.g., primigravida, age 16–24 years, employment, being single or separated) could have several explanations. Younger women in their first pregnancy might have had less interaction with reproductive health services. Also, employed women might have more mobility, which increases risk for STI acquisition. We did not identify any risk factors for *M. genitalium* infection, although younger women (16–24 years of age) were at increased risk for >1 of the curable current STIs. Risk factors for STIs in pregnancy reported elsewhere in Papua New Guinea include having >1 lifetime sexual partner, low education level of the woman or her partner, rural location, history of miscarriage or stillbirth, and low socioeconomic status (*20*,*34*).

This study also provides data on BV and VVC; 57.6% of participants had >1 of these infections. VVC can cause extreme discomfort and increase a woman’s risk for postpartum breast candidiasis, which can affect breastfeeding, but VVC is treatable with antimicrobial drugs (*41*). We found a 37.5% prevalence of VVC, higher than the 23% prevalence reported in Papua New Guinea in 1991 (*42*). Comparisons with other LMICs are difficult because of the limited amount of contemporary data (*41*,*43*). We found a 1-in-4 prevalence of BV among pregnant women, higher than the 17.6% prevalence previously reported in Papua New Guinea (*35*), but in keeping with recent global estimates of 23%–29% (*5*). However, our results might underestimate the true prevalence because diagnosis was limited to only women with a Nugent score of 7–10.

In Papua New Guinea, syndromic management of RTIs is common because access to diagnostic services is limited. We confirm previous reports from Papua New Guinea and elsewhere (*28*,*3*^7^) that syndromic management is an inadequate tool to effectively treat RTIs. This approach missed 78.2% of *M. genitalium* infections and 3 of 4 RTIs. Alternative approaches are essential to effectively prevent, detect, and treat RTIs in a cost-effective, feasible manner in resource-constrained settings. Although condoms are available, their use is limited by gender disparities, stigma, and financial barriers (*23*). Improved access to affordable, accurate point-of-care diagnostics would lead to more accessible and appropriate treatment, resulting in improved sexual and reproductive health; the widespread implementation of GeneXpert for tuberculosis diagnosis (*44*) might also increase access to STI diagnosis in Papua New Guinea.

The main limitation of this study is the facility-based recruitment of participants because results might not represent women who do not attend any antenatal clinic. However, routinely collected provincial data for 2015–2017 estimated that 73%–85% of pregnant women attended >1 appointment at an antenatal clinic (*45**,**46*). The number of women who had a point-of-care syphilis test was lower than other tests. These results did not differentiate between active or latent infection; we also were unable to exclude exposure to yaws, which is endemic to Papua New Guinea (*47*). Yaws and syphilis are caused by different subspecies of *T. pallidum* and cannot be distinguished by this test alone. Prevalence of yaws varies widely within Papua New Guinea; estimates for ENB are unavailable, although neighboring New Ireland Province has a 1.8% prevalence of active yaws according to a population-wide survey (*48*).

In conclusion, we provide data on *M. genitalium* prevalence and antimicrobial resistance markers in Papua New Guinea, revealing a high prevalence of infection underrecognized by syndromic management guidelines. This data contributes to the understanding of the global prevalence of this infection among pregnant women. We found that STIs were common among pregnant women; 37.7% of participants had >1 STI at the time of the study. This study also highlights the high prevalence of BV and VVC and confirms that current antenatal screening practices with syndromic management is inadequate. This high prevalence of disease negatively affects sexual and reproductive health. Urgent action towards ensuring access to affordable prevention, diagnosis, and treatment of RTIs in communities in Papua New Guinea and similar settings is essential. This action will be crucial to achieving the sustainable development goal of ensuring universal access to sexual and reproductive health-care services by 2030 (*49*). Expanding treatment access will contribute to improved sexual and reproductive health outcomes for women in Papua New Guinea.

Appendix Further information on *Mycoplasma genitalium* and other reproductive tract infections in pregnant women in Papua New Guinea, 2015–2017.

## References

[1] Newman L, Rowley J, Vander Hoorn S, Wijesooriya NS, Unemo M, Low N, et al. Global estimates of the prevalence and incidence of four curable sexually transmitted infections in 2012 based on systematic review and global reporting. PLoS One. 2015;10:e0143304. 10.1371/journal.pone.0143304 26646541 PMC4672879

[2] World Health Organization. Report on global sexually transmitted infection surveillance, 2018. 2018 [cited 2019 Dec 9]. https://www.who.int/reproductivehealth/publications/stis-surveillance-2018

[3] Rowley J, Vander Hoorn S, Korenromp E, Low N, Unemo M, Abu-Raddad LJ, et al. Chlamydia, gonorrhoea, trichomoniasis and syphilis: global prevalence and incidence estimates, 2016. Bulletin of the World Health Organization. 2019;97:548–62, 62A–62P. 10.2471/BLT.18.228486 PMC6653813 31384073

[4] van de Wijgert JHHM, Jespers V. The global health impact of vaginal dysbiosis. Res Microbiol. 2017;168:859–64. 10.1016/j.resmic.2017.02.003 28257809

[5] Peebles K, Velloza J, Balkus JE, McClelland RS, Barnabas RV. High global burden and costs of bacterial vaginosis: a systematic review and meta-analysis. Sex Transm Dis. 2019;46:304–11. 10.1097/OLQ.0000000000000972 30624309

[6] Cauchie M, Desmet S, Lagrou K. Candida and its dual lifestyle as a commensal and a pathogen. Res Microbiol. 2017;168:802–10. 10.1016/j.resmic.2017.02.005 28263903

[7] Donovan B. Sexually transmissible infections other than HIV. Lancet. 2004;363:545–56. 10.1016/S0140-6736(04)15543-8 14975619

[8] Arol OA, Over M, Manhard L, Holmes KK. Sexually transmitted infections. In: Jamison DT, Breman JG, Measham AR, Alleyne G, Claeson M, Evans DB, et al., editors. Disease control priorities in developing countries. 2nd ed. New York: Oxford University Press; 2006. p. 315

[9] Thwaites A, Flanagan K, Datta S. Non-HIV sexually transmitted infections in pregnancy. Obstetrics, Gynaecol Reprod Med. 2019;29:151–7. 10.1016/j.ogrm.2019.03.001

[10] Martin DH, Manhart LE, Workowski KA. Mycoplasma genitalium From Basic Science to Public Health: Summary of the Results From a National Institute of Allergy and Infectious Disesases Technical Consultation and Consensus Recommendations for Future Research Priorities. J Infect Dis. 2017;216(suppl_2):S427–30. 10.1093/infdis/jix147 28838075 PMC5853892

[11] Lis R, Rowhani-Rahbar A, Manhart LE. *Mycoplasma genitalium* infection and female reproductive tract disease: a meta-analysis. Clin Infect Dis. 2015;61:418–26. 10.1093/cid/civ312 25900174

[12] Sonnenberg P, Ison CA, Clifton S, Field N, Tanton C, Soldan K, et al. Epidemiology of *Mycoplasma genitalium* in British men and women aged 16–44 years: evidence from the third National Survey of Sexual Attitudes and Lifestyles (Natsal-3). Int J Epidemiol. 2015;44:1982–94. 10.1093/ije/dyv194 26534946 PMC4690003

[13] Baumann L, Cina M, Egli-Gany D, Goutaki M, Halbeisen FS, Lohrer G-R, et al. Prevalence of *Mycoplasma genitalium* in different population groups: systematic review andmeta-analysis. Sex Transm Infect. 2018;94:255–62. 10.1136/sextrans-2017-053384 29440466 PMC5969327

[14] Oakeshott P, Hay P, Taylor-Robinson D, Hay S, Dohn B, Kerry S, et al. Prevalence of *Mycoplasma genitalium* in early pregnancy and relationship between its presence and pregnancy outcome. BJOG. 2004;111:1464–7. 10.1111/j.1471-0528.2004.00276.x 15663138

[15] Harrison MA, Harding-Esch EM, Marks M, Pond MJ, Butcher R, Solomon AW, et al. Impact of mass drug administration of azithromycin for trachoma elimination on prevalence and azithromycin resistance of genital *Mycoplasma genitalium* infection. Sex Transm Infect. 2019;95:522–8. 10.1136/sextrans-2018-053938 30981999 PMC6860407

[16] Machalek DA, Tao Y, Shilling H, Jensen JS, Unemo M, Murray G, et al. Prevalence of mutations associated with resistance to macrolides and fluoroquinolones in *Mycoplasma genitalium*: a systematic review and meta-analysis. Lancet Infect Dis. 2020;20:1302–14. 10.1016/S1473-3099(20)30154-7 32622378

[17] The World Bank Group. Papua New Guinea. 2019 [cited 2020 Feb 25]. https://data.worldbank.org/country/papua-new-guinea

[18] National Statistical Office of Papua New Guinea; ICF International, Inc. Papua New Guinea demographic and health survey 2016–18. 2019 [cited 2020 Aug 10]. https://www.nso.gov.pg/census-surveys/demographic-and-health-survey

[19] Robbers G, Vogel JP, Mola G, Bolgna J, Homer CSE. Maternal and newborn health indicators in Papua New Guinea - 2008-2018. Sex Reprod Health Matters. 2019;27:1686199. 10.1080/26410397.2019.1686199 31790637 PMC7888046

[20] Vallely LM, Toliman P, Ryan C, Rai G, Wapling J, Tomado C, et al. Prevalence and risk factors of *Chlamydia trachomatis*, *Neisseria gonorrhoeae*, *Trichomonas vaginalis* and other sexually transmissible infections among women attending antenatal clinics in three provinces in Papua New Guinea: a cross-sectional survey. Sex Health. 2016;13:420–7. 10.1071/SH15227 28636866

[21] Mola G, Amoa A, Bagita M, Augerea L, Geita L, O’Connor M. Manual of standard managements in obstetrics and gynaecology for doctors, HEOs and nurses in Papua New Guinea. 7th ed. Port Moresby (Papua New Guinea): World Health Organization; 2016. p. 10, 94.

[22] National Department of Health. Standard treatment guidelines for common illness of adults in Papua New Guinea. 6th ed. Port Moresby (Papua New Guinea): World Health Organization; 2012. p. 41–2.

[23] Vallely A, Page A, Dias S, Siba P, Lupiwa T, Law G, et al. The prevalence of sexually transmitted infections in Papua New Guinea: a systematic review and meta-analysis. PLoS One. 2010;5:e15586. 10.1371/journal.pone.0015586 21203468 PMC3009733

[24] Golden MR, Workowski KA, Bolan G. Developing a public health response to *Mycoplasma genitalium.* J Infect Dis. 2017;216(suppl_2):S420–6. 10.1093/infdis/jix200 28838079 PMC5853686

[25] Jensen JS, Cusini M, Gomberg M, Moi H. 2016 European guideline on *Mycoplasma genitalium* infections. J Eur Acad Dermatol Venereol. 2016;30:1650–6. 10.1111/jdv.13849 27505296

[26] Donders GGG, Ruban K, Bellen G, Petricevic L. Mycoplasma/Ureaplasma infection in pregnancy: to screen or not to screen. J Perinat Med. 2017;45:505–15. 10.1515/jpm-2016-0111 28099135

[27] Deborde M, Pereyre S, Puges M, Bébéar C, Desclaux A, Hessamfar M, et al. High prevalence of *Mycoplasma genitalium* infection and macrolide resistance in patients enrolled in HIV pre-exposure prophylaxis program. Med Mal Infect. 2019;49:347–9. 10.1016/j.medmal.2019.03.007 30914213

[28] Hoffman CM, Mbambazela N, Sithole P, Morré SA, Dubbink JH, Railton J, et al. Provision of sexually transmitted infection services in a mobile clinic reveals high unmet need in remote areas of South Africa: a cross-sectional study. Sex Transm Dis. 2019;46:206–12. 10.1097/OLQ.0000000000000931 30363030

[29] Cohen CR, Nosek M, Meier A, Astete SG, Iverson-Cabral S, Mugo NR, et al. *Mycoplasma genitalium* infection and persistence in a cohort of female sex workers in Nairobi, Kenya. Sex Transm Dis. 2007;34:274–9. 10.1097/01.olq.0000237860.61298.54 16940898

[30] Vandepitte J, Muller E, Bukenya J, Nakubulwa S, Kyakuwa N, Buvé A, et al. Prevalence and correlates of *Mycoplasma genitalium* infection among female sex workers in Kampala, Uganda. J Infect Dis. 2012;205:289–96. 10.1093/infdis/jir733 22102734

[31] Peuchant O, Le Roy C, Desveaux C, Paris A, Asselineau J, Maldonado C, et al. Screening for *Chlamydia trachomatis*, *Neisseria gonorrhoeae*, and *Mycoplasma genitalium* should it be integrated into routine pregnancy care in French young pregnant women? Diagn Microbiol Infect Dis. 2015;82:14–9. 10.1016/j.diagmicrobio.2015.01.014 25753079

[32] Labbé AC, Frost E, Deslandes S, Mendonça AP, Alves AC, Pépin J. *Mycoplasma genitalium* is not associated with adverse outcomes of pregnancy in Guinea-Bissau. Sex Transm Infect. 2002;78:289–91. 10.1136/sti.78.4.289 12181470 PMC1744504

[33] Joshua IB, Passmore PR, Sunderland BV. An evaluation of the Essential Medicines List, Standard Treatment Guidelines and prescribing restrictions, as an integrated strategy to enhance quality, efficacy and safety of and improve access to essential medicines in Papua New Guinea. Health Policy Plan. 2016;31:538–46. 10.1093/heapol/czv083 26378052

[34] Wangnapi RA, Soso S, Unger HW, Sawera C, Ome M, Umbers AJ, et al. Prevalence and risk factors for *Chlamydia trachomatis*, *Neisseria gonorrhoeae* and *Trichomonas vaginalis* infection in pregnant women in Papua New Guinea. Sex Transm Infect. 2015;91:194–200. 10.1136/sextrans-2014-051670 25313204

[35] Badman SG, Vallely LM, Toliman P, Kariwiga G, Lote B, Pomat W, et al. A novel point-of-care testing strategy for sexually transmitted infections among pregnant women in high-burden settings: results of a feasibility study in Papua New Guinea. BMC Infect Dis. 2016;16:250. 10.1186/s12879-016-1573-4 27268218 PMC4895793

[36] Marks M, Kako H, Butcher R, Lauri B, Puiahi E, Pitakaka R, et al. Prevalence of sexually transmitted infections in female clinic attendees in Honiara, Solomon Islands. BMJ Open. 2015;5:e007276. 10.1136/bmjopen-2014-007276 25922103 PMC4420977

[37] Vallely LM, Toliman P, Ryan C, Rai G, Wapling J, Gabuzzi J, et al. Performance of syndromic management for the detection and treatment of genital *Chlamydia trachomatis*, *Neisseria gonorrhoeae* and *Trichomonas vaginalis* among women attending antenatal, well woman and sexual health clinics in Papua New Guinea: a cross-sectional study. BMJ Open. 2017;7:e018630. 10.1136/bmjopen-2017-018630 29288183 PMC5778337

[38] Unger HW, Ome-Kaius M, Wangnapi RA, Umbers AJ, Hanieh S, Suen CSNLW, et al. Sulphadoxine-pyrimethamine plus azithromycin for the prevention of low birthweight in Papua New Guinea: a randomised controlled trial. BMC Med. 2015;13:9. 10.1186/s12916-014-0258-3 25591391 PMC4305224

[39] Peters RPH, Dubbink JH, van der Eem L, Verweij SP, Bos MLA, Ouburg S, et al. Cross-sectional study of genital, rectal, and pharyngeal Chlamydia and gonorrhea in women in rural South Africa. Sex Transm Dis. 2014;41:564–9. 10.1097/OLQ.0000000000000175 25118973

[40] Moodley D, Moodley P, Sebitloane M, Soowamber D, McNaughton-Reyes HL, Groves AK, et al. High prevalence and incidence of asymptomatic sexually transmitted infections during pregnancy and postdelivery in KwaZulu Natal, South Africa. Sex Transm Dis. 2015;42:43–7. 10.1097/OLQ.0000000000000219 25504300

[41] Pappas PG, Kauffman CA, Andes D, Benjamin DK Jr, Calandra TF, Edwards JE Jr, et al.; Infectious Diseases Society of America. Clinical practice guidelines for the management of candidiasis: 2009 update by the Infectious Diseases Society of America. Clin Infect Dis. 2009;48:503–35. 10.1086/596757 19191635 PMC7294538

[42] Klufio CA, Amoa AB, Delamare O, Hombhanje M, Kariwiga G, Igo J. Prevalence of vaginal infections with bacterial vaginosis, *Trichomonas vaginalis* and *Candida albicans* among pregnant women at the Port Moresby General Hospital Antenatal Clinic. P N G Med J. 1995;38:163–71. 9522855

[43] Sobel JD. Vulvovaginal candidosis. Lancet. 2007;369:1961–71. 10.1016/S0140-6736(07)60917-9 17560449

[44] Lavu EK, Johnson K, Banamu J, Pandey S, Carter R, Coulter C, et al. Drug-resistant tuberculosis diagnosis since Xpert^®^ MTB/RIF introduction in Papua New Guinea, 2012-2017. Public Health Action. 2019;9(Suppl 1):S12–8. 10.5588/pha.19.0005 31579644 PMC6735453

[45] Papua New Guinea National Department of Health. Sector performance annual review. Port Moresby (Papua New Guinea): Ministry of Health; 2018.

[46] Papua New Guinea National Department of Health. Sector performance annual review. Port Moresby (Papua New Guinea): Ministry of Health; 2016.

[47] Mitjà O, Marks M, Konan DJP, Ayelo G, Gonzalez-Beiras C, Boua B, et al. Global epidemiology of yaws: a systematic review. Lancet Glob Health. 2015;3:e324–31. 10.1016/S2214-109X(15)00011-X 26001576 PMC4696519

[48] Mitjà O, Godornes C, Houinei W, Kapa A, Paru R, Abel H, et al. Re-emergence of yaws after single mass azithromycin treatment followed by targeted treatment: a longitudinal study. Lancet. 2018;391:1599–607. 10.1016/S0140-6736(18)30204-6 29428183 PMC5920722

[49] United Nations. SDG indicators: global indicator framework for the Sustainable Development Goals and targets of the 2030 Agenda for Sustainable Development. 2021 [cited 2021 Jan 14]. https://unstats.un.org/sdgs/indicators/indicators-list

