# Supplementary material for: Mycoplasma genitalium and Other Reproductive Tract Infections in Pregnant Women, Papua New Guinea, 2015–2017
Source: Emerg Infect Dis. 2021 Mar;27(3):894–904. doi: 10.3201/eid2703.201783 (PMC7920647; doi:10.3201/eid2703.201783)
Supplement: Appendix — Further information on Mycoplasma genitalium and other reproductive tract infections in pregnant women in Papua New Guinea, 2015–2017. [file 20-1783-Techapp-s1.pdf]

Article DOI: https://doi.org/10.3201/eid2703.201783

Mycoplasma genitalium and Other Reproductive Tract Infections in Pregnant Women, Papua New Guinea, 2015–2017

Appendix

Syndromic Management of Vaginal Discharge

As per national guidelines, women reporting current abnormal vaginal discharge received intravaginal nystatin pessaries for 7 days or a single clotrimazole pessary and intravaginal clotrimazole cream for 7 days; 2 g oral amoxycillin; 1 g oral probenecid; 2 tablets of augmentin (500 mg amoxicillin and 125 mg clavulanic acid per tablet); and 1 g oral azithromycin. Those who tested positive for syphilis were treated with 3 weekly doses of 2.4 million units of intramuscular benzathine penicillin. If present at the clinic, the woman's partner was also treated; if not, they were referred to that facility's STI clinic for treatment.

Additional Laboratory Methods

We used GeneXpert because it is becoming more widely available in PNG for tuberculosis testing (*1*). Testing usually occurred within 7 days of sample collection. We communicated results to participants to assist treatment decisions. Other samples were shipped to the Burnet Institute in Melbourne, Australia for further testing. GeneXpert uses 1 cartridge for *Chlamydia trachomatis* and *Neisseria gonorrhoeae* and a different cartridge for *Trichomonas vaginalis*. Because of a shipment delay to ENB of cartridges for *T. vaginalis*, we did not test all women for this pathogen. We determined a woman's lifetime exposure to *Treponema pallidum* using the results of the syphilis test given at the point of care.

Shipped vaginal specimens were stored at the Burnet Institute Melbourne at −20°C or −80°C until all samples were received. After thawing the samples, we removed 450 µL from the 1.0 mL Copan Universal Transport Medium (Copan Diagnostics, Inc.,

https://www.copanusa.com) and extracted genomic DNA using the QIAamp BiOstic Bacteremia DNA kit (QIAGEN, https://www.qiagen.com). We tested the extracted DNA for *Mycoplasma genitalium* using the ResistancePlus MG kit (SpeeDx, https://plexpcr.com) which uses the PlexPCR and PlexPrime technologies (SpeeDx) for concurrent amplification of *M. genitalium* and detection of 5 *M. genitalium* point mutations (A2058G, A2058C, A2058T, A2059G, and A2059C) within the macrolide resistance–determining region of the 23S rRNA gene (*2*). The microscopist at Melbourne Sexual Health Centre diagnosed BV by analyzing Nugent scores (Nugent score of 7–10 indicates BV) and VVC by observing pseudohyphae, budding yeasts, or both. We could not facilitate treatment for women with *M. genitalium* infection, BV, or VVC because these diagnoses were based on stored samples that were analyzed many months after collection.

Exposures and Outcomes

We determined predictors of reproductive tract infection at each participant's first antenatal clinic visit. Exposures of interest included clinic details, such as enrollment clinic and setting; participant characteristics at enrollment, such as age group (categorized as 16–24, 25–34, or ≥35 years of age), gravidity (primigravida or multigravida), marital status (partnered or single/separated), history of ever having used a modern contraceptive (World Health Organization definition: oral contraceptive pills, implants, injectables, female sterilization, male sterilization, intrauterine devices, diaphragm, emergency contraception, male and female condoms) (*3*), vaginal discharge, employment status, education, smoking, alcohol use, mid upper arm circumference (categorized as ≤23 cm or > 23cm), self-reported history of sexually transmitted infection, presence of nitrites on urine dipstick (as a proxy for urinary tract infection), previous pregnancy outcome (miscarriage, livebirth, or stillbirth), hemoglobin level (measured in g/dL) and World Health Organization anemia classification for pregnant women at sea level (mild anemia 10.0–10.9 g/dL; moderate 7.0–9.9 g/dL; severe <7.0 g/dL) (*4*); and household characteristics, such as partner education, polygamy, time traveled to clinic, and household monthly expenditure. Outcomes measured were *M. genitalium*, *C. trachomatis*, *N. gonorrhoeae*, *T. vaginalis*, *T. pallidum* (syphilis), bacterial vaginosis, and vaginal candidiasis.

Data Management

Questionnaire responses were entered by the research officer directly into an electronic tablet using a study-specific questionnaire on the platform Mobile Data Studio version 7.3 (CreativityCorp Pty Ltd, https://www.creativitycorp.com). After each clinical visit, questionnaires were uploaded into a central computer, verified for completeness, and exported from Mobile Data Studio to Microsoft Office Excel (Microsoft, https://www.microsoft.com). GeneXpert (Cepheid, https://www.cepheid.com) laboratory results were uploaded by the study data manager on a weekly basis and results checked for accuracy and exported. Laboratory results generated in Melbourne for *M. genitalium*, bacterial vaginosis, and vulvovaginal candidiasis were exported to Microsoft Excel (Microsoft). All Excel files were provided to the first author and uploaded into STATA version 15.0 (StataCorp LLC., https://www.stata.com). The databases were verified and cleaned using STATA 15.0 and merged into 1 database for analysis. All data was stored on password-protected computers accessible only to research personnel.

Calculation for *M. genitalium* Prevalence in East New Britain (ENB) Province.

Recruitment occurred over a period of 26 months and 3 weeks (116.14 weeks [813 days] during March 16, 2015–June 6, 2017. Midpoint was 13.3 months (58.1 weeks [406 days]) after recruitment began, so April 25, 2016.

The population of ENB at the 2011 census was 328,369 (taken July 10, 2011), an annual growth rate of 3.6% since the previous census in 2000. In 2011 in Papua New Guinea, there was a total of 3,497,244 women, of which 1,932,533 (55.3%) were of reproductive age (15–49 years of age). The province of ENB had 159,609 women in 2011. We estimated the number of women of reproductive age (15–49 years) in ENB at the 2011 census as 159,609 × 0.553 = 88,263.8 women. In total, 4 years, 9 months, and 2 weeks (or 4.769 years) elapsed between the July 2011 census and recruitment midpoint. We estimated the number of women 15–49 years of age in ENB at study mid-point as the 2011 census count plus the estimated population growth since census, i.e., 88,263.8 + (88,263.8 × 4.769 × 0.036) = 103,417.278. We found a 12.5% (95% CI 10.0–15.3) estimated population prevalence for *M. genitalium*. We therefore estimated the

number of *M. genitalium* infections among women of childbearing age in ENB 2016 as 103,417.278 × 0.125 = 12,927.159 (95% CI 10,341.728–15,822.843).

References

1. Lavu EK, Johnson K, Banamu J, Pandey S, Carter R, Coulter C, et al. Drug-resistant tuberculosis diagnosis since Xpert® MTB/RIF introduction in Papua New Guinea, 2012-2017. Public Health Action. 2019;9:S12–8. PubMed https://doi.org/10.5588/pha.19.0005

2. Sweeney EL, Trembizki E, Bletchly C, Bradshaw CS, Menon A, Francis F, et al. Levels of *Mycoplasma genitalium* antimicrobial resistance differ by both region and gender in the state of Queensland, Australia: implications for treatment guidelines. J Clin Microbiol. 2019;57:e01555-18. PubMed https://doi.org/10.1128/JCM.01555-18

3. Donders GGG, Ruban K, Bellen G, Petricevic L. Mycoplasma/ureaplasma infection in pregnancy: to screen or not to screen. J Perinat Med. 2017;45:505–15. PubMed https://doi.org/10.1515/jpm-2016-0111

4. Deborde M, Pereyre S, Puges M, Bébéar C, Desclaux A, Hessamfar M, et al. High prevalence of *Mycoplasma genitalium* infection and macrolide resistance in patients enrolled in HIV pre-exposure prophylaxis program. Med Mal Infect. 2019;49:347–9. PubMed https://doi.org/10.1016/j.medmal.2019.03.007

Appendix Table 1. Sociodemographic characteristics and obstetric history of pregnant women tested for reproductive tract infections, East New Britain, Papua New Guinea, 2015–2017*

			Testing									
	Total at ANC1		*Mycoplasma genitalium*		*Chlamydia trachomatis* and *Neisseria gonorrhoeae*		*Trichomonas vaginalis*		Syphilis		Bacterial vaginosis and vaginal candidiasis	
Characteristics	no. (%)	Data NA	n (%)	Data NA	n (%)	Data NA	n (%)	Data NA	n (%)	Data NA	n (%)	Data NA
Total	699 (100.0)		625 (100.0)		641 (100.0)		581 (100.0)		437 (100.0)		653 (100.0)	
Sociodemographic												
Enrollment clinic												
St. Mary's Hospital Vunapope	184 (26.3)		157 (25.1)		169 (26.4)		164 (28.2)		89 (20.4)		161 (24.7)	
Nonga General Hospital	83 (11.9)		81 (13.0)		73 (11.4)		64 (11.0)		68 (15.6)		80 (12.3)	
Kerevat Rural Hospital	125 (17.9)		114 (18.2)		118 (18.4)		90 (15.5)		86 (19.7)		123 (18.8)	
Napapar Health Centre	158 (22.6)		136 (21.8)		141 (22.0)		131 (22.5)		101 (23.1)		152 (23.3)	
Paparatava Health Centre	149 (21.3)		137 (21.9)		140 (21.8)		132 (22.7)		93 (21.3)		137 (21.0)	
Clinic administration												
Government	208 (29.8)		195 (31.2)		191 (29.8)		154 (26.5)		154 (35.2)		203 (31.1)	
Catholic Health Services	491 (70.2)		430 (68.8)		450 (70.2)		427 (73.5)		283 (64.8)		450 (68.9)	
Location												
Urban	342 (48.9)		293 (46.9)		310 (48.4)		295 (50.8)		190 (43.5)		313 (47.9)	
Rural	357 (51.1)		332 (53.1)		331 (51.6)		286 (49.2)		247 (56.5)		340 (52.1)	
Mean age, y (SD); range	26.8 (5.6); 16–49	9	26.8 (5.5); 16–49	9	26.8 (5.6); 16–49	7	26.9 (5.6); 17–49	7	26.7 (5.6); 17–49	7	26.7 (5.5); 16–49	9
Age, y		7		7		6		6		5		7
16–24	275 (39.7)		243 (39.3)		256 (40.3)		228 (39.7)		180 (41.7)		257 (39.8)	
25–34	334 (48.3)		302 (48.9)		302 (47.6)		278 (48.4)		204 (47.2)		315 (48.8)	
≥35	83 (12.0)		73 (11.8)		77 (12.1)		69 (12.0)		48 (11.1)		74 (11.5)	
Highest level of education completed		1		1		1		1				1
Primary (grade 8 or less)	325 (46.6)		302 (48.4)		301 (47.0)		274 (47.2)		204 (46.7)		305 (46.8)	
High school (grade 9,10)	177 (25.4)		151 (24.2)		160 (25.0)		138 (23.8)		112 (25.6)		165 (25.3)	
Secondary, vocational, or tertiary	196 (28.1)		171 (27.4)		179 (28.0)		168 (29.0)		121 (27.7)		182 (27.9)	
Employment status												
Unemployed	531 (76.0)		475 (76.0)		488 (76.1)		443 (76.2)		339 (77.6)		492 (75.3)	
Employed in paid work or student	168 (24.0)		150 (24.0)		153 (23.9)		138 (23.8)		98 (22.4)		161 (24.7)	
Province of birth												
East New Britain	578 (82.7)		518 (82.9)		529 (82.5)		479 (82.4)		362 (82.8)		539 (82.5)	
Other Province	121 (17.3)		107 (17.1)		112 (17.5)		102 (17.6)		75 (17.2)		114 (17.5)	
Religion		1		1		1		1		1		1
Catholic	345 (49.4)		302 (48.4)		317 (49.5)		281 (48.4)		217 (49.8)		315 (48.3)	
Other	353 (50.6)		322 (51.6)		323 (50.5)		299 (51.6)		219 (50.2)		337 (51.7)	
Marital status		2		2		2		2				2
Married or cohabiting	663 (95.1)		593 (95.2)		608 (95.1)		550 (95.0)		418 (95.7)		620 (95.2)	
Single, separated, or widowed	34 (4.9)		30 (4.8)		31 (4.9)		29 (5.0)		19 (4.3)		31 (4.8)	
Polygamy		37		33		34		31		18		34

Characteristics	Total at ANC1		Testing									
			Mycoplasma genitalium		*Chlamydia trachomatis* and *Neisseria gonorrhoeae*		*Trichomonas vaginalis*		Syphilis		Bacterial vaginosis and vaginal candidiasis	
	no. (%)	Data NA	n (%)	Data NA	n (%)	Data NA	n (%)	Data NA	n (%)	Data NA	n (%)	Data NA
1 wife	583 (88.1)		523 (88.3)		531 (87.5)		483 (87.8)		369 (88.1)		548 (88.5)	
>1 wife	79 (11.9)		69 (11.7)		76 (12.5)		67 (12.2)		50 (11.9)		71 (11.5)	
Median household monthly expenditure, kina (IQR)	150 (50–300)	36	150 (50–300)	35	150 (50–300)	30	150 (60–300)	32	150 (60–300)	17	150 (60–300)	35
Median cost of ANC, kina (IQR)	4 (2–20)	17	4 (2–20)	16	4 (2–20)	15	4 (2–20)	14	4 (2–20)	12	4 (2–20)	15
Family planning												
Has used modern contraception		9		9		9		9		4		9
No	569 (82.5)		510 (82.8)		513 (81.2)		469 (82.0)		361 (83.4)		530 (82.3)	
Yes	121 (17.5)		106 (17.2)		119 (18.8)		103 (18.0)		72 (16.6)		114 (17.7)	
Maternal health parameters at ANC1												
Gravidity		2		2		1		1		2		2
Primigravidae	177 (25.4)		159 (25.5)		166 (25.9)		145 (25.0)		111 (25.5)		160 (24.6)	
Multigravidae (2–4)	384 (55.1)		343 (55.1)		343 (53.6)		318 (54.8)		243 (55.9)		363 (55.8)	
Grandmulti (≥5)	138 (19.8)		123 (19.7)		132 (20.6)		118 (20.3)		83 (19.1)		128 (19.7)	
Abnormal vaginal discharge		1										
At any time in current pregnancy	135 (19.3)		118 (18.9)		129 (20.1)		115 (19.8)		79 (18.1)		128 (19.6)	
Currently	98 (14.0)		118 (18.9)		93 (14.5)		81 (13.9)		59 (13.5)		92 (14.1)	
Smoking		1		1		1		1		1		1
Never smoked	427 (61.2)		384 (61.5)		387 (60.5)		352 (60.7)		268 (61.5)		396 (60.7)	
Stopped when pregnant	241 (34.5)		217 (34.8)		226 (35.3)		204 (35.2)		151 (34.6)		27 (4.1)	
Current smoker	29 (4.2)		23 (3.7)		27 (4.2)		24 (4.1)		17 (3.9)		229 (35.1)	
Previous pregnancy outcomes												
Median age at first pregnancy, y (IQR)	21 (19–24)	7	21 (19–24)	7	21 (19–24)	5	21 (19–24)	5	21 (19–24)	5	21 (19–24)	7
History pregnancy loss†			466 (74.6)		475 (74.1)		436 (75.0)		326 (74.6)		491 (75.2)	
Miscarriage	46 (8.8)		44 (9.4)		43 (9.1)		39 (8.9)		29 (8.9)		1 (0.2)	
Abortion	1 (0.2)		1 (0.2)		1 (0.2)		1 (0.2)		0		43 (8.8)	
Stillbirth	16 (3.1)		14 (3.0)		16 (3.4)		14 (3.2)		5 (1.5)		15 (3.1)	
Partner details												
Partner's employment status		18		18		17		14		10		18
Unemployed	269 (39.5)		240 (39.5)		249 (39.9)		229 (40.4)		165 (38.6)		247 (38.9)	
Employed in paid work	411 (60.4)		367 (60.5)		375 (60.1)		338 (59.6)		262 (61.4)		388 (61.1)	
Partner attending ANC1		3		3		3		2		2		4
No	571 (82.0)		518 (83.3)		529 (82.9)		477 (82.4)		363 (83.4)		532 (82.0)	
Yes	123 (17.7)		104 (16.7)		109 (17.1)		102 (17.6)		72 (16.6)		117 (18.0)	

*Values are no. (%) except as indicated. ANC1, first antenatal clinic visit; NA, not available.

†Out of 520 women who had a previous pregnancy. Totals to the right are out of women who had a previous pregnancy and indicated testing.

Appendix Table 2. Relationships between current sexually transmitted infections in pregnant women, East New Britain, Papua New Guinea, 2015–2017*

		Coinfection status, no. (%)		Secondary infection			
Primary infection	Total	Monoinfection	Coinfection	*M. genitalium*	*C. trachomatis*	*N. gonorrhoeae*	*T. vaginalis*
Mycoplasma genitalium	78	32 (41.0)	28 (35.9)	–	20	6	13
Chlamydia trachomatis	122	36 (29.5)	66 (54.1)	20	–	25	36
Neisseria gonorrhoeae	35	4 (11.4)	28 (80.0)	6	25	–	8
Trichomonas vaginalis	117	56 (47.9)	44 (37.6)	13	36	8	–

*Totals do not equal sum of coinfections because some women had multiple infections and not all women received testing for all infections. Sum of mono- and coinfections does not equal infection totals because some women had only 1 test done; whether these women had a mono- or coinfection is unknown.

Appendix Table 3. Relationship between bacterial vaginosis and current sexually transmitted infections in pregnant women, East New Britain, Papua New Guinea, 2015–2017*

		Coinfection status, no. (%)		Infection			
Status	Total	Monoinfection	Coinfection	*Mycoplasma genitalium*	*Chlamydia trachomatis*	*Neisseria gonorrhoeae*	*Trichomonas vaginalis*
Bacterial vaginosis	170	74 (43.5)	69 (40.6)	21	41	13	21

*Totals do not equal sum of coinfections because some women had multiple infections and not all women received testing for all infections. Sum of mono- and coinfections does not equal infection totals because some women had only 1 test done; whether these women had a mono- or coinfection is unknown.

Appendix Table 4. Sensitivity and specificity of clinical symptoms as a marker of RTIs in pregnant women, East New Britain, Papua New Guinea, 2015–2017*

	Diagnostic question					
	Question as per syndromic management			Alternative question		
	Do you currently have any abnormal vaginal discharge?			Have you experienced any abnormal vaginal discharge earlier in the pregnancy or now?†		
Category	p value‡	Sensitivity; specificity	NPV; PPV	p value‡	Sensitivity; specificity	NPV; PPV
Reproductive tract infection						
Mycoplasma genitalium	0.63	15.4 (8.2–25.3); 86.6 (83.5–89.4)	14.1 (7.5–23.4); 87.8 (84.7–90.4)	0.48	21.8 (13.2–32.6); 81.5 (78–84.7)	14.4 (8.6–22.1); 88 (84.8–90.7)
Chlamydia trachomatis	0.08	19.7 (13–27.8); 86.7 (83.4–89.5)	25.8 (17.3–35.9); 82.1 (78.6–85.2)	0.06	26.2 (18.7–35); 81.3 (77.6–84.5)	24.8 (17.6–33.2); 82.4 (78.8–85.6)
Neisseria gonorrhoeae	0.35	20 (8.4–36.9); 85.8 (82.7–88.5)	7.5 (3.1–14.9); 94.9 (92.7–96.6)	0.40	25.7 (12.5–43.3); 80.2 (76.8–83.3)	7 (3.2–12.8); 94.9 (92.6–96.6)
Trichomonas vaginalis	0.05	19.7 (12.9–28); 87.5 (84.1–90.3)	28.4 (18.9–39.5); 81.2 (77.5–84.5)	<0.01	29.1 (21–38.2); 82.5 (78.8–85.9)	29.6 (21.4–38.8); 82.2 (78.4–85.6)
Bacterial vaginosis	1.00	14.1 (9.3–20.3); 85.9 (82.5–88.9)	26.1 (17.5–36.3); 73.9 (70.1–77.5)	0.88	20 (14.3–26.8); 80.5 (76.7–84.0)	26.6 (19.1–35.1); 74.1 (70.1–77.8)
Vulvovaginal candidiasis	<0.01	18.8 (14.1–24.2); 88.7 (85.2–91.6)	50 (39.4–60.6); 64.5 (60.3–68.4)	<0.01	25.7 (20.4–31.7); 84.1 (80.1–87.5)	49.2 (40.3–58.2); 65.3 (61.1–69.4)
Multiple infections						
≥1 current RTI§	0.01	15.6 (11.9–19.9); 93.3 (87.3–97.1)	87.1 (76.1–94.3); 27.7 (23.4–32.4)	<0.01	22.5 (18.2–27.3); 89.3 (82.3–94.2)	85.7 (76.8–92.2); 28.7 (24.2–33.6)
≥1 current STI¶	0.22	15.8 (10.9–22); 88 (83.8–91.5)	44.6 (32.3–57.5); 63.2 (58.4–67.9)	0.15	23 (17.1–29.7); 82.5 (77.7–86.6)	44.2 (34–54.8); 63.8 (58.9–68.6)
≥1 cervical infection#	0.79	14.5 (9.5–20.7); 86.4 (82.7–89.5)	30 (20.3–41.3); 71.4 (67.2–75.4)	0.85	19.9 (14.1–26.8); 80.8 (76.7–84.5)	29.5 (21.2–38.8); 71.5 (67.1–75.5)
≥1 vaginal infection**	<0.01	17.7 (13.9–22); 93.3 (88.6–96.5)	84.2 (74–91.6); 35.9 (31.5–40.5)	<0.01	25.1 (20.8–29.9); 90 (84.7–94.0)	83.5 (75.2–89.9); 37.4 (32.8–42.2)
≥1 GeneXpert diagnosed infection††	0.02	19.4 (13.8–26.1); 88.1 (84.4–91.2)	43.6 (32.4–55.3); 69.8 (65.4–73.9)	<0.01	27.4 (21–34.7); 83 (78.8–86.7)	43.2 (33.9–53); 70.8 (66.3–75)
Any 2 current STIs	0.03	22.7 (13.8–33.8); 86.7 (83.6–89.3)	17.9 (10.8–27.1); 89.7 (86.9–92.1)	0.03	29.3 (19.4–41); 81.4 (78–84.5)	16.8 (10.8–24.3); 90 (87.1–92.4)

*Symptoms refer to abnormal vaginal discharge only (not genital warts, ulcers, or sores). Values expressed are no. (95% CI). BV, bacterial vaginosis; CT, *Chlamydia trachomatis*; MG, *Mycoplasma genitalium*; NG, *Neisseria gonorrhoeae*; NPV, negative predictive value; PPV, positive predictive value; RTI, reproductive tract infection; STI, sexually transmitted infection; TV, *Trichomonas vaginalis*; VVC, vulvovaginal candidiasis.
†1 woman who responded yes to the second question had a missing response to the first question.
‡p value derived from Pearson's χ^2 test.
§Current RTIs include MG, CT, NG, TV, BC, and VVC (syphilis not included).
¶Current STIs include MG, CT, NG, and TV (syphilis not included).
#Cervical infections include MG, CT, and NG.
**Vaginal infections include BV, TV, and VVC.
††GeneXpert diagnosed infections include CT, NG, and TV (Cepheid, https://www.cepheid.com).

Appendix Table 5. Univariable analysis of factors associated with current curable sexually transmitted infections in pregnant women, East New Britain, Papua New Guinea, 2015–2017*

	Infection, OR (95% CI); p value				
Characteristic	*Mycoplasma genitalium*	*Chlamydia trachomatis*	*Neisseria gonorrhoeae*	*Trichomonas vaginalis*	>1 current infection
Enrollment clinic					
Vunapope	Referent	Referent	Referent	Referent	Referent
Nonga	0.67 (0.29–1.59); 0.365	0.95 (0.48–1.87); 0.878	2.44 (0.82–7.23); 0.108	0.82 (0.4–1.7); 0.595	0.78 (0.42–1.47); 0.443
Kerevat	0.86 (0.42–1.76); 0.679	0.58 (0.3–1.09); 0.092	1.02 (0.32–3.29); 0.977	0.65 (0.33–1.29); 0.222	0.5 (0.27–0.91); 0.024
Napapar	0.76 (0.38–1.53); 0.444	0.99 (0.57–1.71); 0.974	1.2 (0.41–3.51); 0.737	1.06 (0.61–1.83); 0.846	0.59 (0.34–1); 0.05
Paparatava	0.99 (0.51–1.91); 0.972	0.8 (0.45–1.41); 0.434	1.58 (0.57–4.36); 0.377	0.83 (0.47–1.47); 0.525	0.81 (0.49–1.35); 0.42
Clinic administration					
Government	Referent	Referent	Referent	Referent	Referent
Catholic Health Services	1.18 (0.7–1.99); 0.542	1.31 (0.84–2.05); 0.235	0.81 (0.39–1.65); 0.555	1.33 (0.82–2.16); 0.241	1.3 (0.86–1.98); 0.215
Location					
Urban	Referent	Referent	Referent	Referent	Referent
Rural	0.97 (0.61–1.57); 0.916	0.75 (0.5–1.11); 0.154	1.43 (0.71–2.86); 0.315	0.75 (0.5–1.13); 0.173	0.88 (0.61–1.27); 0.503
Age, y					
>35	Referent	Referent	Referent	Referent	Referent
25–34	0.81 (0.37–1.79); 0.609	2.66 (1.02–6.95); 0.045	1.16 (0.24–5.46); 0.855	2.04 (0.88–4.71); 0.096	1.84 (0.94–3.6); 0.076
16–24	1.32 (0.6–2.87); 0.487	5.31 (2.06–13.71); 0.001	3.7 (0.85–16.07); 0.081	2.88 (1.25–6.66); 0.013	3.12 (1.58–6.14); 0.001
Gravidity					
Multigravidae	Referent	Referent	Referent	Referent	Referent
Primigravidae	1.18 (0.69–2); 0.549	3.01 (1.99–4.55); <0.001	5.42 (2.66–11.03); <0.001	1.78 (1.14–2.76); 0.01	2.07 (1.37–3.14); 0.001
Highest level of education completed					
Primary (grade 8 or less)	Referent	Referent	Referent	Referent	Referent
High school (grade 9,10)	0.97 (0.53–1.77); 0.919	0.85 (0.51–1.41); 0.529	1.51 (0.67–3.42); 0.318	1.1 (0.67–1.81); 0.698	1.33 (0.84–2.11); 0.22
Secondary, vocational, or tertiary	1.11 (0.64–1.94); 0.707	1.23 (0.78–1.94); 0.381	1.22 (0.53–2.81); 0.64	0.76 (0.46–1.26); 0.285	1.11 (0.72–1.72); 0.636
Employment status					
Unemployed	Referent	Referent	Referent	Referent	Referent
Employed	0.87 (0.49–1.54); 0.626	1.36 (0.88–2.12); 0.17	2.24 (1.11–4.51); 0.025	1.07 (0.67–1.72); 0.769	1.34 (0.88–2.06); 0.172
Time to clinic, min					
<25	Referent	Referent	Referent	Referent	Referent
>25	1.4 (0.81–2.42); 0.23	1.19 (0.75–1.88); 0.45	1.07 (0.5–2.31); 0.855	1.07 (0.68–1.69); 0.771	1.06 (0.7–1.62); 0.779
Mean household monthly expenditure, kina					
<50	Referent	Referent	Referent	Referent	Referent
50–150	1.16 (0.61–2.19); 0.656	1.4 (0.81–2.42); 0.233	1.05 (0.39–2.79); 0.924	1.15 (0.64–2.05); 0.642	1.19 (0.71–2.01); 0.507
150–300	0.87 (0.44–1.71); 0.682	1.14 (0.64–2.04); 0.65	1.18 (0.44–3.14); 0.741	1.33 (0.74–2.37); 0.335	1.18 (0.7–2); 0.527
>300	0.75 (0.35–1.59); 0.449	1.02 (0.55–1.9); 0.939	0.77 (0.25–2.42); 0.66	0.95 (0.51–1.78); 0.882	1.15 (0.66–1.99); 0.622
Marital status					
Married or cohabiting	Referent	Referent	Referent	Referent	Referent
Single, separated, or widowed	1.1 (0.37–3.23); 0.868	2.49 (1.16–5.34); 0.02	1.2 (0.27–5.24); 0.809	4.71 (2.2–10.06); <0.001	1.86 (0.8–4.31); 0.147
Polygamy					
1 wife	Referent	Referent	Referent	Referent	Referent
>1 wife	0.65 (0.27–1.56); 0.332	1.35 (0.75–2.41); 0.32	0.7 (0.21–2.34); 0.558	1.55 (0.85–2.81); 0.152	1.39 (0.79–2.47); 0.256
Smoking history					
Never smoked	Referent	Referent	Referent	Referent	Referent

Characteristic	Infection, OR (95% CI); p value *Mycoplasma genitalium*	*Chlamydia trachomatis*	*Neisseria gonorrhoeae*	*Trichomonas vaginalis*	>1 current infection
Current or past smoker	1.44 (0.89–2.32); 0.137	1.12 (0.75–1.67); 0.579	1.3 (0.66–2.59); 0.447	1.19 (0.79–1.8); 0.396	0.94 (0.65–1.37); 0.76
Alcohol consumption in previous month					
No	Referent	Referent	Referent	Referent	Referent
Yes	1.11 (0.37–3.28); 0.853	0.77 (0.29–2.05); 0.597	1.27 (0.29–5.61); 0.756	1.14 (0.45–2.91); 0.785	1.21 (0.52–2.8); 0.658
Current abnormal vaginal discharge					
No	Referent	Referent	Referent	Referent	Referent
Yes	1.18 (0.61–2.29); 0.628	1.59 (0.95–2.66); 0.077	1.51 (0.64–3.56); 0.35	1.71 (1–2.91); 0.049	1.39 (0.82–2.35); 0.225
History of abnormal vaginal discharge during current pregnancy					
No	Referent	Referent	Referent	Referent	Referent
Yes	1.23 (0.69–2.2); 0.483	1.54 (0.97–2.44); 0.064	1.4 (0.64–3.06); 0.401	1.94 (1.22–3.09); 0.005	1.4 (0.89–2.2); 0.147
Previous STI					
No	Referent	Referent	Referent	Referent	Referent
Yes (self-reported)	0.83 (0.4–1.74); 0.628	0.68 (0.35–1.29); 0.234	0.61 (0.18–2.03); 0.417	1.25 (0.7–2.21); 0.447	0.95 (0.56–1.62); 0.859
Have used modern contraception					
Yes	Referent	Referent	Referent	Referent	Referent
No	1.92 (0.89–4.11); 0.095	1.66 (0.94–2.93); 0.081	1.83 (0.63–5.3); 0.263	1.61 (0.89–2.9); 0.114	1.63 (0.98–2.7); 0.06
Mid-upper arm circumference, cm					
>23	Referent	Referent	Referent	Referent	Referent
<23	1.13 (0.6–2.15); 0.704	0.74 (0.4–1.36); 0.335	1.02 (0.38–2.71); 0.967	0.83 (0.45–1.53); 0.542	0.7 (0.41–1.2); 0.19
Height, cm	0.99 (0.95–1.04); 0.726	1.01 (0.97–1.05); 0.664	0.98 (0.92–1.05); 0.566	0.99 (0.96–1.03); 0.785	0.99 (0.96–1.02); 0.471
Hemoglobin					
HemocueResult†	0.96 (0.81–1.14); 0.628	1.02 (0.88–1.18); 0.815	1.16 (0.89–1.52); 0.258	0.96 (0.83–1.12); 0.631	0.98 (0.85–1.13); 0.757
Not anemic	Referent	Referent	Referent	Referent	Referent
Mild anemia	0.67 (0.29–1.54); 0.346	1.12 (0.56–2.26); 0.746	1.58 (0.52–4.77); 0.421	1.72 (0.85–3.46); 0.131	0.89 (0.47–1.69); 0.733
Moderate anemia	0.94 (0.47–1.85); 0.851	1.39 (0.76–2.55); 0.286	1.05 (0.37–2.97); 0.928	1.27 (0.67–2.38); 0.465	1.04 (0.61–1.77); 0.888
Severe anemia	1.6 (0.51–5.03); 0.422	0.7 (0.19–2.62); 0.595	1	0.57 (0.12–2.7); 0.477	1.23 (0.42–3.58); 0.703
Urine nitrites‡					
Negative	Referent	Referent	Referent	Referent	Referent
Trace	0.54 (0.13–2.34); 0.411	0.93 (0.31–2.79); 0.897	0.8 (0.1–6.14); 0.83	0.4 (0.09–1.76); 0.227	0.44 (0.14–1.34); 0.148
Positive	1.28 (0.58–2.84); 0.542	1.79 (0.95–3.39); 0.073	1.47 (0.5–4.34); 0.489	1.59 (0.81–3.13); 0.179	1.48 (0.76–2.87); 0.252
Fever during pregnancy					
No	Referent	Referent	Referent	Referent	Referent
Yes (before ANC1)	1.22 (0.72–2.08); 0.457	0.87 (0.55–1.39); 0.561	0.88 (0.39–1.99); 0.763	1.71 (1.1–2.66); 0.017	1.21 (0.79–1.86); 0.379

*ANC1, first antenatal clinic; OR, odds ratio.
†HemoCue, https://www.hemocue.com?
‡Indicates urinary tract infection.
